# Identification of a sex pheromone of the chrysanthemum lace bug *Corythucha marmorata* (Hemiptera: Tingidae)

**DOI:** 10.1038/s41598-017-06783-y

**Published:** 2017-08-04

**Authors:** Kisaki Watanabe, Nobuhiro Shimizu

**Affiliations:** Faculty of Bioenvironmental Science, Kyoto Gakuen University, 1-1 Nanjo, Sogabe, Kameoka 621-8555 Japan

## Abstract

Although the nymphs of *Corythucha marmorata* form clusters on the undersides of host plant leaves, as frequently observed for Hemiptera, the adults are scattered in the vicinity of the nymph population. By investigating the biological activities of volatile secretions from the adult, we found that the secretions activated male mounting behaviour. A chemical analysis revealed that borneol was a common component of the secretions from both sexes. The absolute configuration of the natural product was the (+)-enantiomer of borneol and the optical isomer was undetectable. Although (+)-borneol showed significant sex pheromone activity against males, the antipode (−)-borneol also induced sex pheromone activity, albeit only slightly. Males may not have a strict identification mechanism based on stereochemistry. To verify the origin of this sex pheromone, we analysed the components of the essential oil of the leaves of *Solidago canadensis* L. (Compositae: Asteraceae), a host plant; bornyl acetate was detected to be a major component. The plant-produced bornyl acetate had different stereochemistry from the sex pheromone. The results suggested that the adults do not utilise the secondary metabolites of plants but biosynthesise this sex pheromone themselves. This is the first report on sex pheromone identification in Tingidae.

## Introduction

Tingidae (Hemiptera) are distributed throughout the world, and many cluster in the mesophyll tissue on the undersides of host plant leaves. In Japan, *Corythucha marmorata*, *C*. *ciliata*, and *Dulinius conchatus* have been confirmed as invasive species^1^. *C*. *marmorata* was discovered as an agricultural pest infesting Asteraceae plants in southern Canada and the US^2^. In Japan, *C*. *marmorata* has expanded its distribution area since its occurrence was first confirmed in 2000 on goldenrod, *Solidago canadensis*, in Nishinomiya City, Hyogo Prefecture^3–5^. The main hosts of *C*. *marmorata* are Asteraceae weeds such as *S*. *canadensis* and *Ambrosia artemisiifolia*; however, the damage has now spread to sunflower, sweet potato, and eggplant.

When one of *C*. *marmorata* nymphs in a group is crushed with a needle, the other nymphs around it exhibit an escape action. Similar escape behaviour can also be reproduced using a nymph’s secretions from dorsal abdominal glands. Geraniol, which is a nymph-specific component, has been identified as an alarm pheromone in *C*. *marmorata* as well as in *C*. *ciliata* and *Gargaphia solani* ^6–9^ and affects both adults and nymphs in *C*. *marmorata* ^6^. In contrast, to the best of our knowledge, pheromones that affect the mounting behaviour of the adults have not been reported in Tingidae. In Miridae, closely related to the Tingidae, females of many species release long-range sex pheromones to attract conspecific males^10, 11^. To date, the sex pheromones produced by females of 16 species have been identified^12^.

When we observed the mating behaviour of *C*. *marmorata* adults, we noticed that the male first mounted on the female’s back, shook his body up and down, and then moved his genitals toward the female to begin mating. The hexane extract of the adults significantly activated the mounting behaviour of males; therefore, the aim of this study was to identify the sex pheromone in these volatile secretions from the exocrine glands of adults. To determine whether the sex pheromone is biosynthesised by the adults themselves or derived from the plants they eat, the components of the essential oil of the leaves of one of its host plants, *S*. *canadensis*, were obtained by steam distillation and identified.

## Results

**Figure 1:**
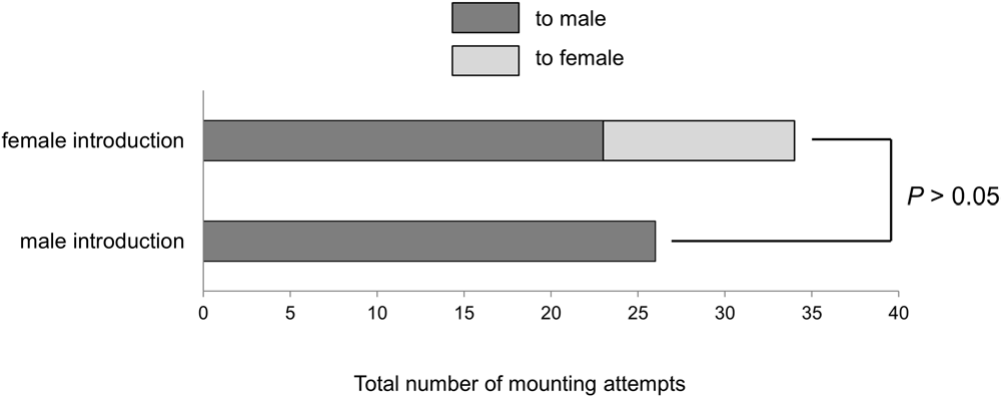
The total number of mounting attempts by males to each introduced sex (*N* = 20). Statistical differences were evaluated using a Mann–Whitney *U* test (*P* < 0.05).

When a female was introduced into the assay chamber, previously isolated and rested males showed mounting attempts (Fig. 1). At that time, the conditioned males also showed mounting attempts to individuals of the same sex. When introduction of a male was tested in the same manner, mounting attempts were observable between the introduced male and conditioned males and/or among the conditioned males. These results indicate that males cannot discriminate between males and females for the mounting behaviour. The total number of male mounting attempts after introducing a female was slightly more than after introducing a male, with no significant difference (*P* > 0.05, Fig. 1).

**Figure 2:**
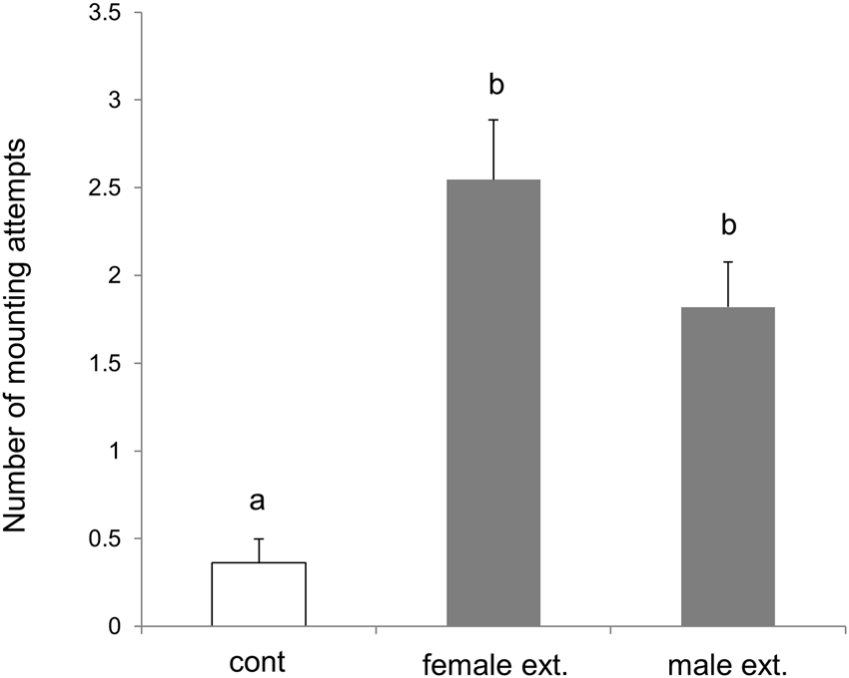
Results of tests for mounting behaviour in males using hexane extracts from females and males. Bars represent the means ± SE. (*N* = 10). Statistical differences were evaluated using a Steel–Dwass multiple comparison test (*P* < 0.05).

In the biological tests, we used the mounting behaviour of a male on the back of another male while shaking his body up and down as the standard mating behaviour to evaluate pheromone activity. The hexane extract at a dose of 0.1 female equivalent activated mounting behaviour among males. The frequency of mounting behaviour in 5 min was significantly higher among tested males than in control (*P* < 0.05, Fig. 2). The mounting behaviour of males was also reproducible by exposure to the male hexane extract (0.1 male equivalent). The frequency of mounting behaviour was significantly higher than that of control (*P* < 0.05, Fig. 2). There is no significant difference in the frequency of mounting behaviour between the hexane extract from both sexes (*P* > 0.05, Fig. 2).

**Figure 3:**
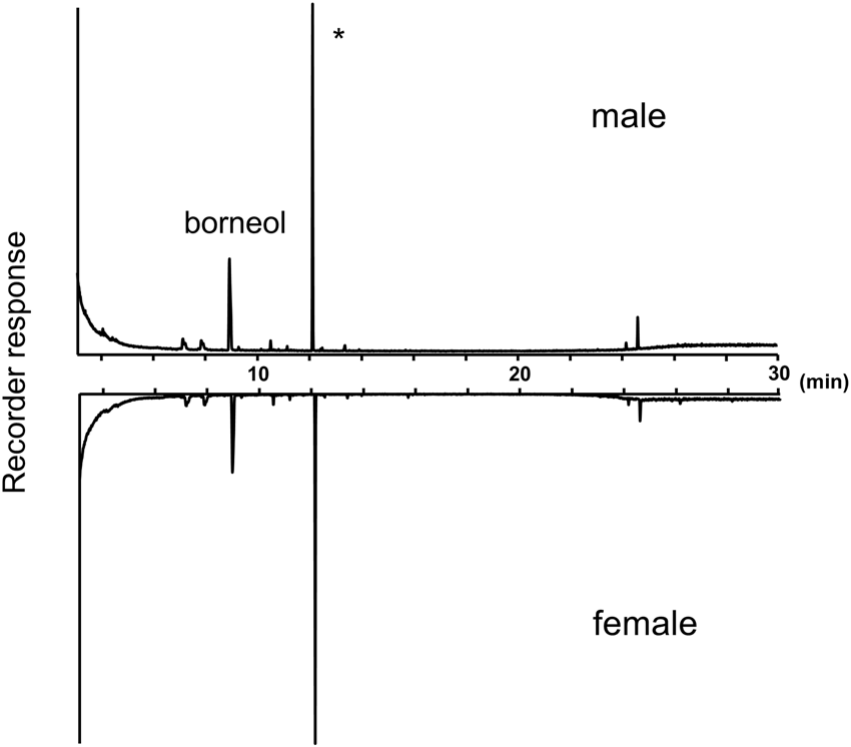
Typical gas chromatogram of hexane extracts from male and female *Corythucha marmorata*. An *asterisk* (*) indicates the tetradecane internal standard in the hexane extracts.

Gas chromatography-mass spectrometry (GC-MS) analysis of the hexane extract from both sexes of *C*. *marmorata* indicated that females and males produce a specific component with a retention time of 8.769 min (Fig. 3) that is not present in the hexane extract from nymphs^6^. No qualitative sexual differences were identified in the GC profiles. The MS spectrum of the adult-specific compound showed a base ion peak at *m*/*z* 95 (100%) and characteristic ion peaks at *m*/*z* 152 (M^+^, 1), 139 (7), 136 (6), 121 (7), 110 (20), 67 (9), 55 (9), and 41 (11). Searching the Agilent NIST05 Mass Spectral Library for the fragmentation pattern indicated that the pattern was consistent with that of borneol. Thus, because the retention time and MS spectrum of commercially available borneol exactly matched those of the natural product, we concluded that the adult secretory component is borneol. Quantitative analysis using GC-MS indicated that 12.9 ± 1.6 ng and 11.5 ± 1.2 ng of borneol was extracted from females and males, respectively. Thus, the females produced approximately 1.1-times more borneol than males, although this difference was not statistically significant (*P* > 0.05, *N* = 10).

**Figure 4:**
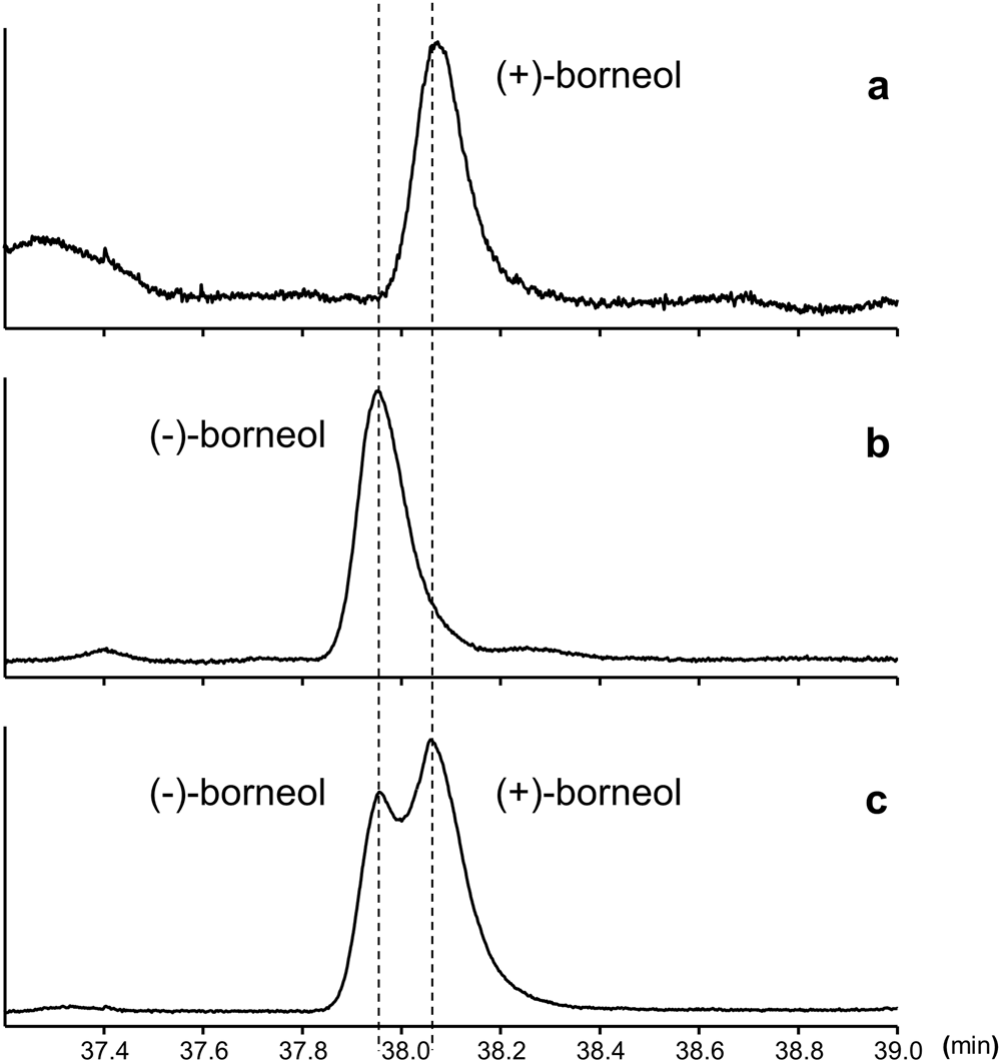
Gas chromatograms of (**a**) (+)-borneol extracted from adults, (**b**) (−)-borneol derived from host plant-produced bornyl acetate, and (**c**) authentic racemate of (−)- and (+)-borneol.

The absolute configuration of borneol was determined using a GC system equipped with a chiral column. Although the authentic racemate was not completely separated, we were still able to determine the absolute configuration because the retention times of the enantiomers were different; the retention time of the (−)-enantiomer was 37.95 min, whereas that of the (+)-enantiomer was 38.07 min (Fig. 4C). The hexane extracts from five adults were pooled and used for a single analysis. The retention time of the natural product was consistent with that of the (+)-enantiomer (Fig. 4A). We also performed chromatographic analysis of the natural product and racemate, which confirmed an increase in the peak of the (+)-enantiomer.

**Figure 5:**
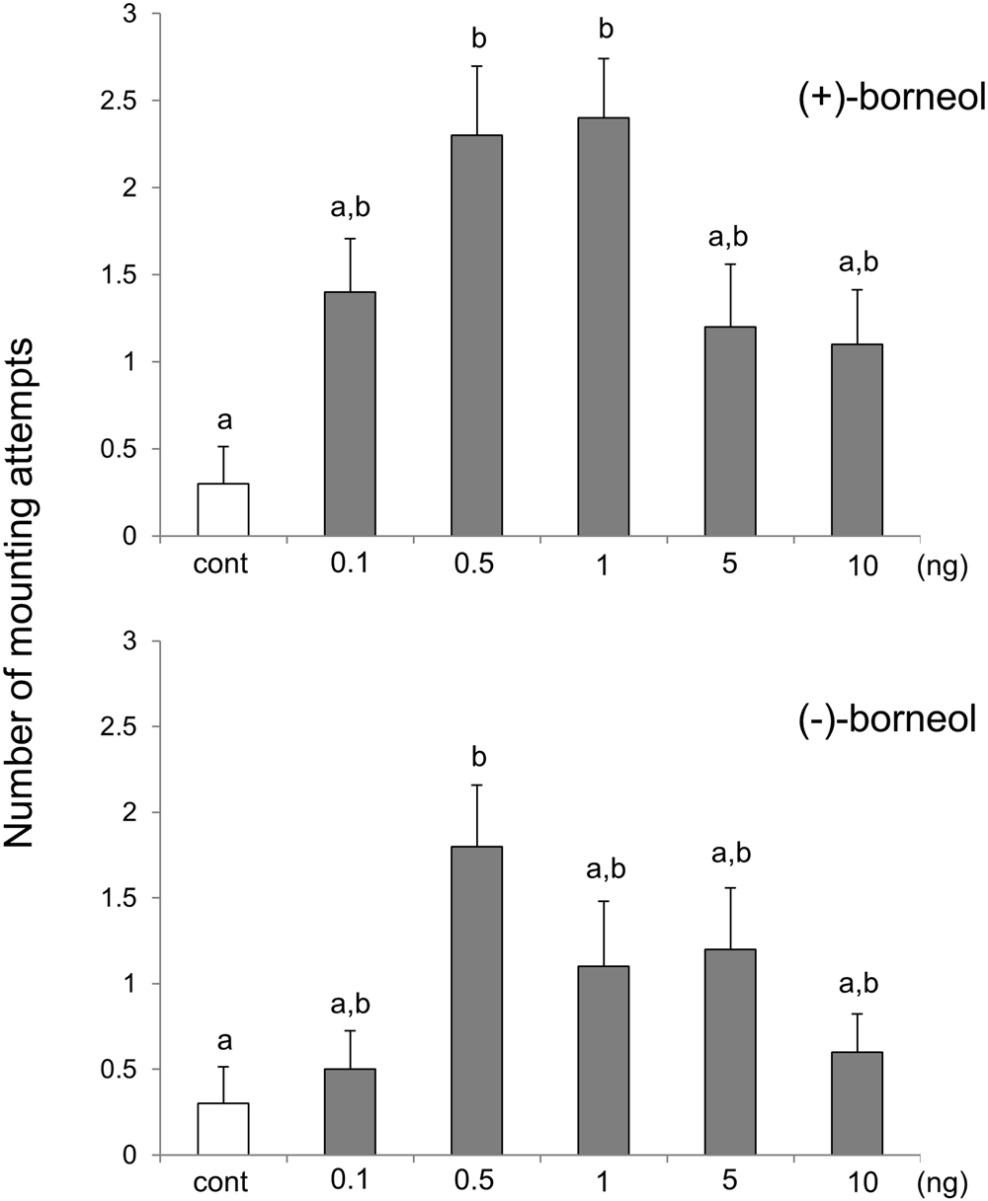
Results of tests for mounting behaviour in males using authentic (+)-borneol and (−)-borneol. Bars represent the means ± SE. (*N* = 10). Statistical differences were evaluated using a Steel–Dwass multiple comparison test (*P* < 0.05).

We evaluated the sex pheromone activity in males using (+)-borneol. Significant activation of mounting behaviour was observed in the presence of 0.5 and 1 ng (+)-borneol (Fig. 5). In addition, mounting behaviour was slightly induced at a low (+)-borneol concentration of 0.1 ng, suggesting that the males are very sensitive to this sex pheromone. On the other hand, when the dosage was greater than the optimum amount (0.5 and 1 ng), the sex pheromone activity was reduced.

Thus, (+)-borneol was identified as a sex pheromone specific for males. Next, we performed the biological test with (−)-borneol. Although the activation of mounting behaviour with (−)-borneol was weaker than that with (+)-borneol, we still observed sex pheromone activity at a dose of 0.5 ng (Fig. 5). A slight activation of mounting behaviour was also observed with 1 and 5 ng of (−)-borneol; however, this activation was not significantly different from that observed in the control group. Because males also show the sex pheromone activity in the presence of (−)-borneol, which has a different stereochemistry from the sex pheromone, we concluded that they do not have a strict mechanism for molecular identification.

To verify whether the (+)-borneol that we identified as a sex pheromone is derived from components of the plants that are eaten by the insect, the essential oil components were obtained from the leaves of *S*. *canadensis* by steam distillation and analysed using GC-MS. Bornyl acetate (20.2%) and germacrene D (54.0%) were detected as major components, whereas α-pinene (2.4%), sabinene (4.2%), limonene (9.1%), δ-elemene (2.5%), β-elemene (1.6%), *trans*-caryophyllene (2.2%), and elemol (3.8%) were detected as minor components. The components other than bornyl acetate, α-pinene, and limonene were tentatively identified through a comprehensive search of the Agilent NIST05 Mass Spectral Library and the results of previous analytical studies of these plant components^13–16^.

The aim of this study was to determine the absolute configuration of bornyl acetate, which is an acetyl product of the sex pheromone borneol. The essential oil components of goldenrod leaf extract were purified using silica gel column chromatography. The bornyl acetate was then eluted in 10% ethyl acetate/hexane with the aim of analysing it by chiral GC. However, the authentic racemate was not separated. Therefore, bornyl acetate was deacetylated to produce borneol. Borneol was then used for chiral GC analysis. The results indicated that borneol from the leaves of goldenrod existed as the (−)-enantiomer; thus, it had a different absolute configuration from the sex pheromone derived from adults (Fig. 4).

## Discussion

Some species of Tingidae are known to use the alarm pheromone for conspecific communication^6–9^; however, the sex pheromone related to sexual excitation has not yet been elucidated. The presented results demonstrated that the volatile secretions from the adult play a crucial role in the mounting behaviour of *C*. *marmorata* males. We observed that males demonstrated the mounting behaviour on live insects of both sexes (Fig. 1). Therefore, we hypothesised that the hexane extract of secretions from both sexes contains a sex pheromone that activates male mounting behaviour. This assumption is consistent with the fact that, as shown in Fig. 2, males demonstrated mounting attempts induced not only by the hexane extract from females but also from males. Females were never observed to respond to the hexane extract from both sexes. In the case of the rice leaf bug *Trigonotylus caelestialium*, the components of the female sex pheromone were common in the body extracts of both sexes^17^. However, traps baited with *T*. *caelestialium* males did not attract conspecific males^18^.

We observed that the hexane extract activated male mounting behaviour on females as well as males. These results indicated that males cannot discriminate between males and females for mounting. In the case of the stink bug *Piezodorus hybneri*, sex pheromone released by males elicits homosexual behaviour in males^19^. This behaviour, which was first observed in aposematic beetles, *Lycus loripes*, strongly suggests that the semiochemicals are involved in sexual recruitment^20^. Homosexual behaviour has been reported in most insect orders^21^, and Scharf and Martin reviewed evidence of such behaviour in more than 100 species of insects and arachnids^22^. Compared with flying insects using sex attractant pheromones, lace bugs form colonies on the backs of leaves, and their ability to scatter over long distances seems to be restricted. In the natural condition of relatively high density, such erroneous sex recognition by active males may occur^22^. The bioassay method conducted in the present study might induce the remarkable mounting behaviour between males using female sex pheromone^19^ or keeping males in isolation^23^, all of which are unusual for field conditions.

For arachnids, some examples of ixodid ticks have been reported in which an attractant sex pheromone, 2,6-dichlorophenol, is produced by both sexes^24, 25^; however, the influence of the pheromones on the males remains unknown. In the case of astigmatid mites, the female sex pheromones are distributed in both sexes in different amounts, even during the nymphal stages^26^. The differences in the content ratios of female sex pheromones between males and females function to aid males in recognising conspecific females. In the species used in the present study, the female sex pheromone is distributed in nearly same amounts between both sexes (Fig. 3). If the compound functions more favourably like a defensive substance for insect’s survival, the compound would be widely distributed in both sexes in exchange for the lack of the male’s ability to discriminate conspecific females from males. Blum suggested the possibility that sex pheromones in arthropods have evolved from defensive secretions^27^. In some species of Miridae, chemicals from the scent glands may have a dual function of serving as defence at high doses and as pheromone components to attract members of the opposite sex at lower doses^28^. Further studies should be performed to evaluate whether the female sex pheromone of *C*. *marmorata* functions as defensive allomones against predators.

In this study, the absolute configuration of the sex pheromone borneol secreted by *C*. *marmorata* was elucidated as (+)-isomer (Fig. 4). Using GC with chiral capillary column, the natural borneol was confirmed to be enantiomerically pure with no trace of antipode contamination. The biological tests revealed that (+)-borneol was active at 0.5–1 ng doses, whereas (−)-borneol was active at 0.5 ng dose (Fig. 5). (+)-Borneol induced the activity, and the (−)-isomer exhibited a weaker activity than the (+)-isomer. Although the activity of the sex pheromone depends on the chirality, it may be reasonable to assume that males cannot clearly discriminate between the absolute configurations of the sex pheromone borneol. As a similar example, it is known that both enantiomers of the alarm pheromone 4-methyl-3-heptanone exhibit alarm pheromone activity in the leaf-cutting ant *Atta texana* ^29^. The (*S*)-isomer is approximately 100 times more active than (*R*)-isomer against the worker ants.

**Figure 6:**
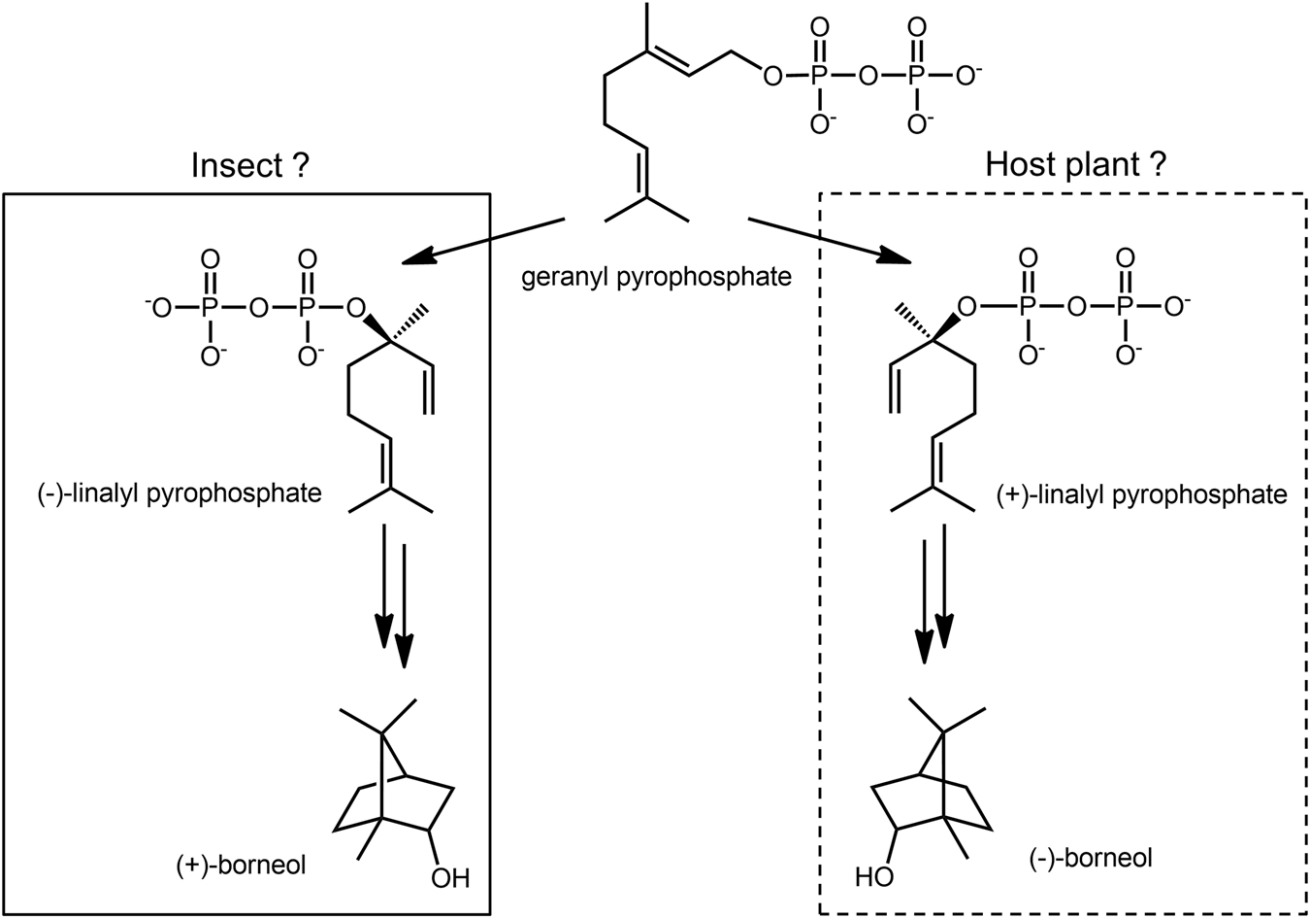
Proposed biosynthetic pathway of (+)- and (−)-borneol from geranyl pyrophosphate. Figure modified from Croteau, R.B. *et al*., 1985.

To verify the origin of this sex pheromone, we analysed the components of the essential oil of the leaves of *S. canadensis*, a host plant; bornyl acetate was detected to be a major component. The plant-produced bornyl acetate had different stereochemistry from the sex pheromone (Fig. 4). Therefore, these findings suggest that Tingidae adults do not simply use hydrolysed bornyl acetate derived from plants as a sex pheromone; they biosynthesise borneol with a different absolute configuration. Because this Tingidae species is also a sunflower parasite, we analysed the volatile components of sunflower leaves by GC-MS. We detected the presence of bornyl acetate and germacrene D, as observed in goldenrod (data not shown). Although the absolute configuration of bornyl acetate in sunflower leaves was not examined, goldenrod and sunflower, which both belong to Asteraceae, were found to contain the same major volatile components. In plants, (+)-borneol has been reported to be biosynthesised from geranyl pyrophosphate via (−)-linalyl pyrophosphate, as shown in Fig. 6 ^30^. To the best of our knowledge, there has been no report on biosynthetic enzymes for cyclic monoterpene borneol production in insects. This suggests that a completely different enzyme from that found in plants may be involved in this biosynthesis. In future studies, we will examine the conversion reaction of the substrate geranyl pyrophosphate to borneol using crude enzyme prepared from adults.

## Materials and Methods

### Insects

*C*. *marmorata* that were fed on the leaves of *S*. *canadensis* growing naturally around the campus of Kyoto Gakuen University were collected, adult males and females were sexed under a stereomicroscope, then separated and reared in 3.5 L plastic containers with fresh leaves of goldenrod at room temperature. The leaves were replaced three times per week. Although the age and mating status of adults were not distinguished, adults that passed more than two days after their final moult were used for all of the experiments.

### Biological tests

All biological tests were conducted at 28 °C ± 2 °C with ambient humidity in the laboratory. The sexes of adults were separated according to the form of the abdomen and external genital. The abdomen of female is thick and round, and the end of the male’s abdomen is rather tapered, the genital can be observed under a stereomicroscope. Previously isolated and rested males initiated homosexual mounting behaviour when exposed to filter paper (5 mm square) soaked in an extract from females. The extract was prepared by dipping ten females into 200 μl of hexane for 3 min. Based on these observations, the bioassay was designed to examine whether males could discriminate between males and females. A single, fresh goldenrod leaf was placed into a transparent glass Petri dish (diameter = 6.0 cm, height = 2.0 cm), and 5 previously isolated males were moved onto the leaf using a brush. The dishes were covered with a net of fine meshes and maintained for 30–60 min until all insects had stopped moving. To examine the behavioural responses of males to live insects, a single male or female was gently introduced to the leaf in the dishes. The number of males mounting the back of another male or female while shaking their bodies up and down was then counted for 5 min. The biological tests were conducted 20 times for each treatment. A Mann–Whitney *U* test was performed to compare the total number of mounting attempts by males to each introduced sex.

Because males respond to live insects of both sexes, the behavioural responses of males to the hexane extracts prepared from adults of both sexes were investigated. A 100-μl glass insert was inserted into a 1.5-ml GC glass vial, and one insect was placed in the glass insert using a brush. The secretions from exocrine glands were extracted by dipping the entire body for 3 min in hexane (5 μl). A piece of filter paper (5 mm square), impregnated with the hexane extracts of secretions (0.1 adult equivalent) or candidate compound (0.1, 0.5, 1, 5, or 10 ng) dissolved in hexane (1 μl) was placed on the leaf without causing any disturbance (e.g. vibration). Hexane was used as a control. The biological tests were conducted 10 times for each treatment. A Steel–Dwass non-parametric multiple-group comparison test was performed to compare the number of mounting attempts by males.

### Chemical analyses

All chemicals used were of reagent grade. Column chromatography was performed on a Wakosil silica gel C-200 with the specified solvents. GC-MS was conducted using a Network GC System (6890N; Agilent Technologies Inc.) coupled with a mass-selective detector (5975 Inert XL; Agilent Technologies Inc.) operated at 70 eV using an HP-5MS capillary column (0.25-mm i.d. × 30 m, 0.25-μm film thickness; Agilent Technologies Inc.). Helium was used as the carrier gas (flow rate = 1.00 ml/min) in splitless mode. The temperature was increased from 60 °C (2 min) to 290 °C at a rate of 10 °C/min and then maintained at 290 °C for 5 min. GC was performed using an Agilent Technologies 6890N instrument equipped with a flame ionisation detector using an InertCap CHIRAMIX capillary column (0.25-mm i.d. × 30 m, 0.25-μm film thickness; GL Sciences Inc.). Helium was used as the carrier gas (flow rate = 1.00 ml/min) in splitless mode. The temperature was increased from 50 °C to 180 °C at a rate of 5 °C/min and then maintained at 180 °C for 30 min.

### Quantitative and stereochemical determination of borneol

The hexane extracts of secretions from *C*. *marmorata* adults of both sexes were independently quantitatively analysed using GC-MS. A 100-μl glass insert was inserted into a 1.5-ml GC glass vial, and one insect was placed in the glass insert using a brush. The secretions were extracted by adding 5 μl of hexane solution containing tetradecane (5 ng) as an internal standard. After 3 min, 1 μl of the extract was injected into the GC-MS instrument using a 10-μl micro-syringe. The analysis was repeated ten times for both male and female extracts. A Mann–Whitney *U* test was performed to compare the contents between the two sexes.

The absolute configuration of borneol in the hexane extracts was determined using a GC system equipped with an InertCap CHIRAMIX capillary column. A racemic mixture of borneol was prepared by mixing equal amounts of authentic (−)- and (+)-borneol.

### Stereochemical determination of bornyl acetate contained in goldenrod leaves

The components of the essential oil of goldenrod leaves were extracted from fresh, intact leaves (83 g) of goldenrod via steam distillation. Ethyl acetate was then added to the aqueous solution containing the essential oil components, and the organic phase was separated and dried over anhydrous sodium sulphate. After drying, the solvent was concentrated using an evaporator to obtain the components of the essential oil (687 mg).

One part of the obtained essential oil components was diluted with hexane and used for GC-MS analysis. Subsequently, 500 mg of the essential oil components was applied to a silica gel column (25 g) and sequentially eluted with hexane, 10% ethyl acetate/hexane, 20% ethyl acetate/hexane, 40% ethyl acetate/hexane, and ethyl acetate (250 ml of each solution).

Bornyl acetate (15 mg) eluted in 10% ethyl acetate/hexane, and the purified bornyl acetate was deacetylated to produce borneol. The absolute configuration of borneol was determined using the chiral GC procedure described previously. Deacetylation was performed under the following procedure: bornyl acetate (15 mg) was dissolved in anhydrous diethyl ether (0.5 ml), and one microspatula of LiAlH_4_ was added at 0 °C; the sample was warmed to room temperature and reacted for 20 min; the reaction solution was cooled with ice, and the reaction was stopped through the addition of water; ether was added; and the organic layer was washed with brine and dried over anhydrous sodium sulphate. This process produced an ether solution containing borneol, which was used directly for GC analysis.
